# Pet Dogs and Children’s Health: Opportunities for Chronic Disease Prevention?

**DOI:** 10.5888/pcd12.150204

**Published:** 2015-11-25

**Authors:** Anne M. Gadomski, Melissa B. Scribani, Nicole Krupa, Paul Jenkins, Zsolt Nagykaldi, Ardis L. Olson

**Affiliations:** Author Affiliations: Melissa B. Scribani, Nicole Krupa, Paul Jenkins, Research Institute, Bassett Medical Center, Cooperstown, New York; Zsolt Nagykaldi, University of Oklahoma Health Sciences Center, Oklahoma City, Oklahoma; Ardis L. Olson MD, Dartmouth Medical School, Lebanon, New Hampshire.

## Abstract

**Introduction:**

Positive associations between having a pet dog and adult health outcomes have been documented; however, little evidence exists regarding the benefits of pet dogs for young children. This study investigates the hypothesis that pet dogs are positively associated with healthy weight and mental health among children.

**Methods:**

This cross-sectional study accrued a consecutive sample of children over 18 months in a pediatric primary care setting. The study enrolled 643 children (mean age, 6.7 years); 96% were white, 45% were female, 56% were privately insured, and 58% had pet dogs in the home. Before an annual visit, parents of children aged 4 to 10 years completed the DartScreen, a comprehensive Web-based health risk screener administered using an electronic tablet. The screener domains were child body mass index (BMI), physical activity, screen time, mental health, and pet-related questions.

**Results:**

Children with and children without pet dogs did not differ in BMI (*P* = .80), screen time of 2 hours or less (*P* = 0.99), or physical activity (*P* = .07). A lower percentage of children with dogs (12%) met the clinical cut-off value of Screen for Child Anxiety and Related Disorders (SCARED-5) of 3 or more, compared with children without dogs (21%, *P* = .002). The mean SCARED-5 score was lower among children with dogs (1.13) compared with children without dogs (1.40; *P* = .01). This relationship was retained in multivariate analysis after controlling for several covariates.

**Conclusions:**

Having a pet dog in the home was associated with a decreased probability of childhood anxiety. Future studies need to establish whether this relationship is causal and, if so, how pet dogs alleviate childhood anxiety.

## Introduction

**Figure 1:**
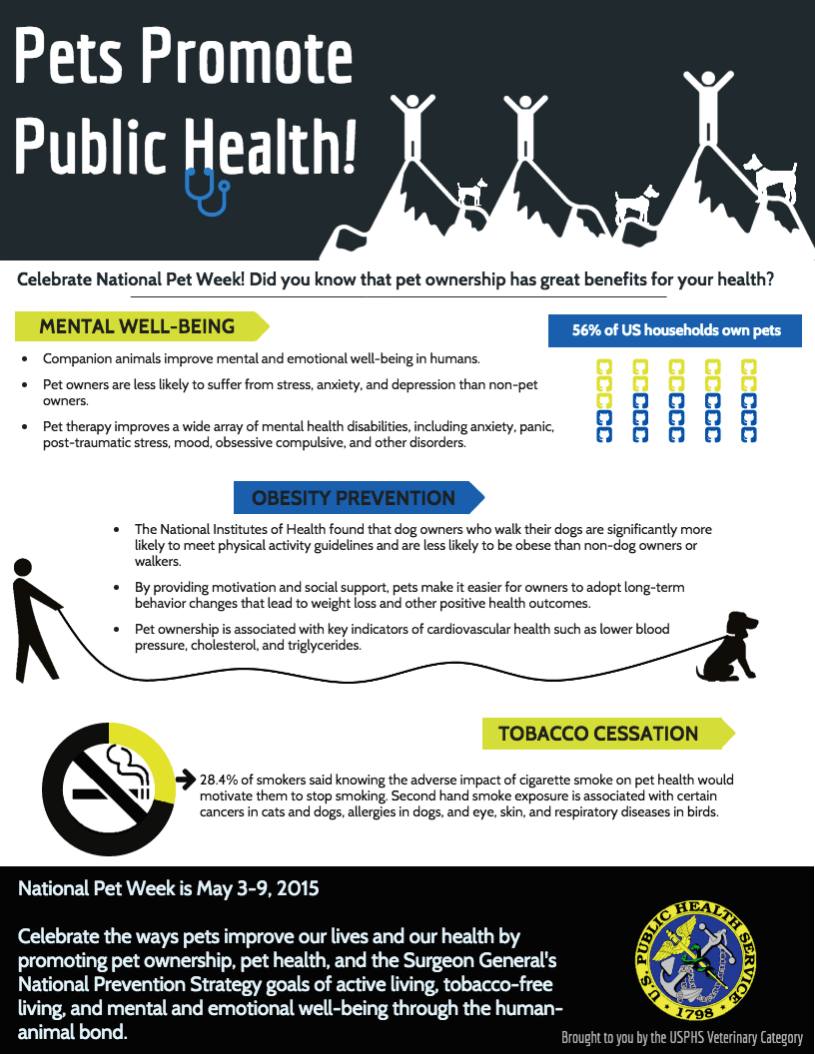
US Public Health Service flyer, “Pets Promote Health,” describing benefits of pet ownership.

Childhood mental illness and obesity are significant public health problems in the United States (1,2). Because both conditions start in childhood, preventive and early intervention approaches are needed. Pet dogs have been linked with varied physical and mental health benefits for adults (3,4), benefits that are promoted by the US Public Health Service (USPHS) (Figure 1). Although dog ownership may improve adult physical activity, body weight, and mental health (5,6), less is known about the relationship between pet dogs and children’s health. In Australia and the United Kingdom, dog ownership was associated with increased accelerometer-measured physical activity among children aged 5 to 12 years (7–9) and a lower likelihood of overweight or obesity among children aged 5 to 6 years (10). In those countries, promoting walking and active play with a dog is an effective strategy to increase children’s physical activity. Such studies have not been done in the United States, so more evidence is needed to support this as a US strategy.

Children may interact with dogs in other ways that may benefit them. From a mental health standpoint, children aged 7 to 8 often ranked pets higher than humans as providers of comfort and self-esteem and as confidants (11,12). Animal-assisted therapy (AAT) with dogs affects children’s mental health and developmental disorders by reducing anxiety and arousal or enhancing attachment (13). Because dogs follow human communicative cues, they may be particularly effective agents for children’s emotional development (14). Despite the evidence for the therapeutic effects of AAT for certain childhood conditions, little evidence is available for primary care providers to use when counseling parents regarding the benefits of pet dogs for young children.

**Figure 2:**
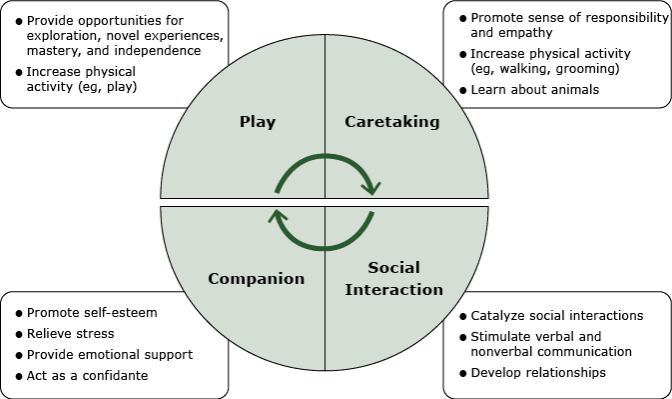
Model for how pet dogs may influence the physical and mental health of children aged 4 to 10 years. The model summarizes study findings regarding how pet dogs promote children’s behavioral and emotional development, mental health (3,4, 11–13,30), and physical activity (6–10,26).

Promoting children’s behavioral and emotional competence is an effective strategy to prevent mental, emotional, and behavioral disorders during adulthood (15). If exposure to pet dogs during childhood is inversely related to mental health problems, positive child­–­dog interactions could prevent the evolution of these problems into full-fledged disorders during adolescence or later life. Studies support this possibility (Figure 2). Our study investigated the hypothesis that pet dogs are positively associated with healthy weight and mental health among children.

## Methods

This cross-sectional study was conducted at a general pediatric clinic in an academic medical center at the hub of a not-for-profit rural health network in Upstate New York. From July 2012 through December 2013, we consecutively recruited parents of children, aged 4 to 10 years who came to clinic for their annual check-ups. Over the 18 months, we consecutively enrolled 643 children who were 96% white, 45% female, and 56% privately insured. One child per family was eligible; ill or developmentally disabled children were excluded. Before the doctor’s check-up the parent completed the DartScreen, a comprehensive Web-based child health screener using an electronic tablet (16,17) . A research assistant entered the age, sex, height, and weight measured by the nurse.

The DartScreen includes questions about somatic and mental health concerns, nutrition, physical activity, screen time, general health, anxiety, parental depression, and whether or not the child has emotional difficulties or difficulties with attention, behavior, or getting along with others (17). A pet module added to the end of the DartScreen was automatically triggered by a question about having pets in the home: “Do you live with a pet in your home?” If the parent selected “yes,” the screener branched to the kind of pet. If dog was selected, the screener branched to more detailed questions about the dog, including duration of exposure to the pet dog in the child’s lifetime and time spent being physically active with the dog. If the family had more than one dog, the parent was prompted to think of the dog that the child spent the most time with. The Flesch-Kincaid Reading Ease score for the pet module was 91 and the grade level was 3.

Mental health measures were 4 widely used and validated assessment tools used for screening but not for formal diagnosis. DartScreen automatically coded responses and calculated scores for the validated scales it contains. All parents completed the SCARED-5, a 5-item scale adapted from the Screen for Child Anxiety and Related Disorders, a screening tool for childhood anxiety disorders validated in both psychiatric (18) and primary care settings (19,20). The abbreviated SCARED-5 has shown psychometrics similar to the full 41-item SCARED screening tool, which measures general anxiety, separation anxiety, social phobia, school phobia, and physical symptoms of anxiety. In addition to analyzing the mean SCARED-5 score, the proportion of children meeting the SCARED-5 clinical score threshold of 3 or more was also analyzed.

For children with reported emotional, attention, or behavioral difficulties, the screener branched to the Strengths and Difficulties Questionnaire (SDQ) Impact Supplement (21) and, if responses were positive, to the Pediatric Symptom Checklist (22).The SDQ inquires whether the child has difficulties in 4 areas (emotion, concentration, behavior, and getting along with others) and whether such difficulties interfere with home life, friendships, classroom learning, and leisure activities. SDQ questions are scored at 3 levels (0, not at all or a little; 1, a medium amount; 2, a great deal) to yield an impairment score of 0 to 10 for unlikely, possible, or probable mental disorder — the higher the score, the more probable a mental disorder (21). The PSC-17, adapted from the Pediatric Symptom Checklist (PSC), assesses psychosocial problems among children and youths aged 6 to 16 years (23). PSC-17 has been validated against structured psychiatric interviews, and includes subscales for internalizing, externalizing, and attention conditions; however, it is less accurate for anxiety disorders (23).

Because a parent’s mental health can affect reporting about the child (24), the DartScreen included the 2-item Patient Health Questionnaire (PHQ-2), which has a sensitivity of 79% and specificity of 86% for any depressive disorder (25). We used the PHQ-2, a commonly used screening test for depression, as a brief measure of parental depressive symptoms. To be at risk for depression, the parent had to have a score of 3 or higher out of a possible 6, a cut point that has been validated.

### Potential confounders

Research on human–animal interactions requires controlling for potential covariates such as age, sex, socioeconomic status (SES), and race/ethnicity, which may account for the differences between pet owners and nonowners (9,12,26). Families who own pets may differ from families who do not have pets by several factors that can also influence a child’s health (26). Among a set of 17 SES indicators, a community's poverty rate was ranked as the leading measure of socioeconomic environment that is a social determinant of health (27). Family income is also significantly related to adolescent mental health, accounting for 28% of the prevalence of DSM IV disorders among adolescents (2). Therefore, we investigated 2 SES proxies: 1) the type of health insurance the child had, and 2) the percentage of the population living below the 2013 poverty level in the child’s residence zip code (28). In our catchment area, this latter indicator ranges from 4% to 61%. Type of health insurance (none, Medicaid, Child Health Plus [New York State Children's Health Insurance Program], or commercial insurance) was not related to the dependent variables. However, poverty level (percentage of population under the poverty threshold by zip code) was related to SDQ impact and PSC-17, so it was used to adjust multivariate analyses.

### Sample size

Using the proportion of overweight or obese children in Australia aged 5 to 6 years who owned a dog (19.8%) and did not own a dog (25.2%) (10), we estimated that the proportion of overweight US children among those with dogs versus those without would be 20% and 25% respectively. Assuming a 1-tailed *α* of .05, a sample of 311 subjects in both of these groups (622 subjects total) provided a power of .80 for the test of the null hypothesis of no difference in proportions. Our target sample size was 650, and our effective sample size was 643 after excluding cases with incomplete data or duplicates.

### Statistical methods

At the conclusion of data collection, data were de-identified before analysis. Univariate comparisons of BMI classes, screen time (dichotomized as ≤2 hours/d vs >2 hours/d), physical activity, SDQ impairment, PSC-17, SCARED-5, and the child’s history of mental disorder were made between the pet dog group and the no pet dog group. Because the distribution of SCARED-5 skewed to the right, this variable was transformed to square-root values before analyses. Mean values and 95% confidence intervals were then back-transformed to display in tables.

Using SAS 9.3 (SAS Institute), continuous variables, such as age, BMI z-score, total SCARED-5 score and total PSC-17 score, were compared between groups by using the *t* test. Categorical variables, such as BMI classification, parent PHQ-2 positivity, SCARED-5 score of 3 or greater, 3-level SDQ impairment, and the child’s history of mental disorder were compared between the pet dog group and the no pet dog group by using the χ^2 ^test.

Multivariate analyses were carried out using multiple linear regression for composite outcomes (SCARED-5 and total PSC-17), and using logistic regression for dichotomous outcomes for SCARED-5 (≥ 3 vs < 3) and SDQ impairment (SDQ ≥2 vs <2). In these multivariate models, we controlled for the child’s sex, the child’s age, the parent PHQ positivity, and percentage of population under the poverty threshold in the child’s zip code.

To further delineate the relationship between specific anxiety elements and dog ownership, we analyzed the 5 screener items of the SCARED-5. The scores for each item were compared between the pet dog group and no pet dog group by using the *t* test.

We also conducted subanalyses to assess the relationship between anxiety and other mental health measures. Specifically, the distribution of 3-level SDQ impairment of function was compared between children with SCARED-5 of 3 or greater and children with SCARED-5 of less than 3 by using χ^2^. The 4-item internalizing subscale of the PSC-17 was also dichotomized at the cut point and compared with SCARED-5 ≥ 3 by using χ^2^.

The Bassett Hospital Institutional Review Committee approved this study on March 19, 2012, and reviews it annually for the life of the study.

## Results

Among the 643 enrolled children, 470 (73%) had a pet, 133 (21%) had no pets, and 40 (6%) were missing all pet data and were assigned to the no-pet-dog group. Among the 470 with pets, 4 were missing data about the type of pet; 3 of these were assigned to the group with pet dogs because they had answered screener questions specific to a pet dog. The remaining subject was assigned as having no pet dog. In the final analysis, 370 (57.5%) children with a pet dog were compared with 273 (42.5%) with no pet dog.

Mothers most often completed the screener (80% mother, 16% father, 4% other). A total of 20 parents scored positively on the PHQ-2 (3.2%), and 14 (2.2%) parents reported family issues they wished to discuss with the provider during the visit. We observed no significant difference in parental PHQ-2 between those with and those without a pet dog (Table 1).

**Table 1:** Summary Statistics for Children’s (N = 643) Health Indicators and Results of Bivariate Tests for Effects of a Pet Dog in the Home

Indicator	n^a^	Sample	Pet Dog in Home (n = 370)	No Pet Dog in home (n = 273)	*P* Value^b^
Female, %	643	45.1	45.1	45.1	.98
Age, mean (95% CI)	643	6.72 (6.55– 6.88)	6.72 (6.50– 6.94)	6.71 (6.46, 6.96)	.94
Poverty level, mean (95% CI)^c^	643	0.15 (0.14–0.15)	0.15 (0.14– 0.16)	0.15 (0.14– 0.15)	.23
Positive on Parent PHQ, %	617	3.2	3.3	3.1	.87
Child history of mental health diagnosis, %	594	7.2	7.5	6.9	.76
PSC-17 score, mean (95% CI)^d^	177	11.75 (10.80– 12.70)	11.55 (10.14– 12.96)	11.99 (10.73– 13.25)	.65
Screen time ≤2 h/d, %	630	54.9	54.9	54.9	.99
BMI *z*-score, mean (95% CI)	640	0.54 (0.46– 0.63)	0.53 (0.42– 0.65)	0.56 (0.43– 0.68)	.80
**BMI^e^, %**					
Normal	423	66.1	65.8	66.5	.80
Overweight	108	16.9	17.7	15.8	
Obese	109	17.0	16.6	17.7	
**SCARED-5 score, mean (95% CI)**					
Mean SCARED-5 (untransformed [raw data])	630	1.24 (1.14– 1.35)	1.13 (1.00– 1.26)	1.40 (1.23– 1.58)	.01
Mean SCARED-5 (transformed)	630	0.74 (0.65– 0.84)	0.65 (0.54– 0.77)	0.89 (0.73– 1.06)	.02
SCARED-5 score ≥3, %	630	15.7	12.0	21.0	.002
**SDQ impact scores, %**					
Normal (SDQ = 0)	551	87.7	89.0	85.9	.33
Borderline (SDQ = 1)	29	4.6	4.7	4.6	
Abnormal (SDQ ≥2)	48	7.6	6.3	9.5	

Abbreviations: BMI, body mass index; CI, confidence interval; PHQ, PSC, Pediatric Symptom Checklist; SCARED, Screen for Child Anxiety and Related Disorders; SDQ, Strengths and Difficulties Questionnaire Impact Supplement.

a The number for covariates. These numbers do not equal 643 in cases where data were missing for the given covariate.

b *P* value is for having a dog versus not having a dog.

c Percentage of population below the poverty level in NY by zip code.

d The screener branched to the Pediatric Symptom Checklist if the SDQ Impact Supplement was positive. Therefore not all children were screened with the PSC.

e We used the CDC definitions for the 3 child BMI classes (normal, overweight, obese): overweight = BMI ≥85th percentile and <95th percentile for children of the same age and sex; obesity = BMI ≥95th percentile for children of the same age and sex).

We found no difference between children with and children without a pet dog in BMI (*P* = .80) or screen time of 2 hours or more (*P* = .99) (Table 1). We also found no difference for physical activity (*P* = .07). Among families with pet dogs, BMI *z*-score was not associated with the parent’s reported time that the child was physically active with the dog (*P* = .15).

Twenty-six children (7.6%) who had pet dogs had a history of a mental disorder, and 15 (7.0%) children with a mental disorder history did not have a pet dog (*P* = .76). The mean PSC-17 score for children with a pet dog was 11.6 versus 12.0 for children without a pet dog (*P* = .65) (Table 1).

Ninety-nine children (15.7%) had a SCARED-5 score of 3 or higher. The mean age for children with a positive SCARED-5 score (6.71 years) was virtually identical to those with a negative SCARED-5 score (6.70 years) ( *P* = .95). Girls had higher mean SCARED-5 scores than did boys; however, there was no difference in percentage scoring SCARED-5 ≥ 3. In univariate analyses, the mean SCARED-5 score was significantly lower among children with a pet dog (1.13 untransformed, 0.65 transformed) compared with children without a dog (1.40 untransformed, 0.89 transformed, *P* = .02) (Table 1). Duration of pet dog exposure (years) in the child’s lifetime was not correlated with the SCARED-5 score (*r* = - 0.087, *P* = .10). Table 2 displays the results for each component of SCARED-5 stratified by pet dog or no pet dog. Significant differences between groups were found for the separation anxiety component (“My child is afraid to be alone in the house”) and social anxiety component (“My child is shy”) favoring pet ownership.

**Table 2:** Screen for Child Anxiety and Related Disorders (SCARED-5) Component Questions Comparing Children With a Pet Dog with Children Without A Pet Dog

Question	Pet Dog in Home, Mean	No Pet Dog in Home, Mean	*P* Value
My child gets really frightened for no reason at all.	0.14	0.20	.07
My child is afraid to be alone in the house.	0.31	0.42	.02
People tell me that my child worries too much.	0.13	0.14	.99
My child is scared to go to school.	0.07	0.06	.64
My child is shy.	0.49	0.60	.01

Among children with a SCARED-5 score of 3 or higher, 18.6% had an SDQ Impairment score of 2 or higher compared with only 5.5% with an SDQ score of 2 or higher among those with a SCARED-5 score lower than 3 (*P* < .001). This finding suggests that the child’s anxiety score was associated with functional impairment. For children with a SCARED-5 score of 3 or higher, 12.5% scored positively on the internalizing subscale of the PSC-17. In contrast, only 1.6% of children who scored lower than 3 on SCARED-5 scored positively on this subscale.

The significant association between pet dog versus no pet dog groups and SCARED-5 score was maintained in a linear regression model controlling for poverty level, parent PHQ positivity, age, and sex (Table 3). This finding was also true in the logistic regression model for children meeting the SCARED-5 score point of 3 or higher. The predicted probability of a SCARED-5 score of 3 or higher was 0.20 for children without pet dogs compared with 0.11 for children with pet dogs. A pet dog in the home was associated with a 9% decreased probability of a SCARED-5 score of ≥ 3 or higher.

**Table 3:** Multivariate Regression Showing Relationship Between Having a Pet Dog in the Home and Child Body Mass Index *Z*-Score and Child Mental Health Indicators, Adjusted for Poverty Level, Parent PHQ Positivity, Age, And Sex

Variable	*β* Coefficient^a^	SE	95% CI	*P* Value
Child BMI *z* score	0.01	0.09	−0.16 to 0.19	.87
SCARED-5	−0.27	0.11	−0.49 to 0.06	.01
PSC-17 score	−0.70	0.89	−2.46 to 1.06	.43
**Variable**	**Odds Ratio^b^**	**SE**	**95% CI**	***P *Value**
SCARED-5 score ≥3	0.49	0.23	0.31 to 0.77	.002
SDQ score ≥2	0.63	0.30	0.35 to 1.15	.13

Abbreviations: BMI, body mass index; CI, confidence interval; PSC-17, Pediatric Symptom Checklist-17; SCARED-5, Screen for Child Anxiety and Related Disorders-5; SDQ, Strengths and Difficulties Questionnaire.

a Linear regression results are shown for BMI z score, SCARED-5 score, and PSC-17 score.

b Logistic regression result is shown for the binary outcomes of SCARED-5 score. <3 vs ≥3. and SDQ score, <2 vs ≥2.

## Discussion

Our study results suggest that children who have a pet dog in the home have a lower anxiety screening score than children who do not. A greater percentage (21%) of children without pet dogs than children with pet dogs (12%) had a SCARED-5 score of 3 or higher, a point at which further assessment is indicated to diagnose anxiety. The anxiety scores in our study were higher for girls than boys as were the social and separation anxiety subfactor distribution. These findings are consistent with those documented in a predominantly white primary care sample of slightly older children (8–12 y) (19). Whereas that study showed no variation with demographic factors, our study found and controlled for an association between SCARED score and poverty level. Despite controlling for age, sex, poverty level, and parent PHQ positivity in our multivariate models, the association between having a pet dog in the home and a lower child anxiety score remained significant. However we observed no difference in body weight, screen time, or physical activity between children with and children without pet dogs in the home.

Because anxiety disorders often start in childhood, often persist into adulthood, and have the longest delays for treatment (eg, age 20–23 y for social and separation anxiety disorders) , addressing subthreshold conditions in primary care settings during childhood is a reasonable target for preventive interventions (2,11,20,21). Our study findings are more relevant to a discussion of subthreshold conditions than of disorders, because this study was of a primary care population as opposed to a population of children with diagnosed mental disorders or a clinically referred population. Only 18% of children who met the SCARED-5 cutoff also met SDQ criteria for probable mental disorder, whereas 82% of children who met the SCARED-5 cutoff did not meet SDQ criteria. This finding is consistent with the relatively high rates of the different types of anxiety among children, including subthreshold anxiety symptoms (2).

Pet dogs could reduce childhood anxiety, particularly social and separation anxiety, by various mechanisms (Figure 2). A pet dog can stimulate conversation, an ice-breaking effect that can alleviate social anxiety via a social catalyst effect (12). Companionship with a pet can alleviate separation anxiety and strengthen attachment (13). Social interaction of humans and dogs may also lead to increased oxytocin levels in both the human and the dog (29). Interacting with a friendly dog also reduces cortisol levels most likely through oxytocin release, which attenuates physiologic responses to stress (29). These hormonal effects may underlie the observed emotional and behavioral benefits of AAT and pet dogs.

The advantage of this study is that it used a real-world setting for data collection, adapted a tablet for in-clinic data collection, and enabled a more comprehensive analysis of the relationship between pet dogs and children’s mental health symptoms while adjusting for several covariates. The study was of children being seen for preventive care, a far larger and more inclusive group of children than those in prior human–animal interaction studies, which focused on children with mental and developmental disorders.

Because this was a cross-sectional study of associations, a correlational study, no cause or effect can be inferred. It may be that less anxious children have pet dogs or pet dogs make children less anxious. To make such inferences, a quasi-experimental design is required in which families who acquire a pet dog are followed longitudinally with a comparison group. Although AAT lends itself to randomized controlled trial design, routine pet exposure does not; however, children spend more time with pets at home than they would with AAT animals. This study does not answer whether pet dogs have direct effects on children’s mental health or whether other factors associated with acquisition of a pet dog benefit their mental health.

This study was limited by parental report. However, parental concerns about emotional and behavioral problems, if carefully elicited, can detect mental health problems among children age 4 years or older (30). Furthermore, in primary care settings, moderate to high concordance between parents’ and children’s reported anxiety scores have been documented (19,20). Given the homogeneity of this study population (96% white), this study requires replication in settings that have more racially and ethnically diverse populations (20).

The USPHS currently promotes pet dogs for improving adult physical and mental health (Figure 1). However, more evidence is needed before promoting interactions between pet dogs and children. Yet, pet dog ownership was associated with a 9% reduction in the probability of a SCARED-5 score of 3 or higher. If this were an effect size, it could provide significant prevention on a population level, assuming a broad reach but only if the relationship is found to be causal. However, a prospective study is required to establish the magnitude of the potential effect as well as causality. Future research should establish the direction of causality, the specificity and magnitude of the effect, and its potential long-term impact on anxiety.
